# Familiarity preferences in zebrafish (*Danio rerio*) depend on shoal proximity

**DOI:** 10.1111/jfb.15963

**Published:** 2024-10-14

**Authors:** William T. Swaney, Caitlyn Ellwood, Joshua P. Davis, Adam R. Reddon

**Affiliations:** ^1^ School of Biological and Environmental Sciences Liverpool John Moores University Liverpool UK

**Keywords:** choice tests, *Danio rerio*, familiarity, shoaling preferences, social behaviour, zebrafish

## Abstract

Individuals of many species prefer to associate with familiar conspecifics from their established social group over unfamiliar conspecifics. Such familiarity preferences are thought to be adaptive and have been documented widely in many social fishes. Zebrafish (*Danio rerio*) are extensively studied, highly social fish that form stable shoals in the wild, however there is only mixed evidence for familiarity preferences in this species. Here, we test how a small variation in experimental design can influence preferences for familiar conspecifics in *D. rerio* by varying the distance between two stimulus shoals of fish in a shoaling choice paradigm. Individual subjects tested for their preference to shoal with familiar or unfamiliar groups of conspecifics showed a preference for familiar fish when the two shoals were 30 cm apart, but not when they were 45 or 60 cm apart. Thus, choice tests can be used to detect familiarity preferences in *D. rerio*, but only when alternate shoals are close together, as increased intershoal distances seemingly prevent subjects from displaying their preference. Longer distances may inhibit preference behavior due to the higher risk of crossing between shoals, alternatively subjects may be unable to reliably discern distinguishing cues of familiarity when the shoals are further apart. Our results demonstrate that while familiarity preferences exist in *D. rerio*, experimental test design is critical for detecting and measuring these successfully.

## 1 INTRODUCTION

Individual social animals often choose to join groups based on properties that enhance the benefits or limit the costs of group living for the individual, and such decision‐making has been well documented in fish. Shoaling fish typically choose larger groups over smaller ones (Hager & Helfman, 1991; Pritchard et al., 2001; Svensson et al., 2000), while phenotypically similar individuals are often preferred to dissimilar ones, choices that are thought to reduce an individual's risk of predation within a group (Ioannou et al., 2011; Krause & Godin, 1995; Landeau & Terborgh, 1986; Rodgers et al., 2010). Individuals will also avoid diseased or parasitized individuals and preferentially associate with healthy ones (Barber et al., 1998; Croft et al., 2011; Tobler & Schlupp, 2008), even avoiding uninfected conspecifics that are merely mounting an immune response (Encel et al., 2023), decisions that would limit social exposure to potential pathogens. Groups exhibiting signs of recent foraging success are also preferred, as shoals of well‐fed individuals have been shown to be favored as social partners over ones composed of hungry fish (Krause et al., 1999).

While these examples of social decision‐making appear to have clear fitness benefits, a more nuanced aspect of group‐choice behavior is the preference for familiar individuals. When two groups are otherwise equivalent but one is made up of familiar individuals and the other of unfamiliar individuals, many fish will preferentially associate with the familiar group (Griffiths & Ward, 2011; Ward et al., 2020). Such familiarity preferences have been documented across a wide range of fish species, inhabiting different environments, with different life histories and varied social organization (Barber & Ruxton, 2000; Courtenay et al., 2001; Jordan et al., 2010; Magurran et al., 1994). Familiarity in this context is not simply that individuals have previously encountered each other, but that they have formed a stable association over time. For example, social familiarity in guppies (*Poecilia reticulata*) takes 12 days to be established, after which individuals will show robust social preferences for familiar group members over novel unfamiliar conspecifics (Griffiths & Magurran, 1997a). This is thought to be due to the time it takes for social and/or dominance relationships to stabilize (Höjesjö et al., 1998; Utne‐Palm & Hart, 2000) and is thus distinct from simple individual recognition in which a previously encountered individual can be distinguished from a novel one (Madeira & Oliveira, 2017; Norton et al., 2019).

Shoaling with familiar conspecifics appears to provide benefits across multiple domains, including predation, foraging, and social advantages. Shoals of familiar individuals have been shown to exhibit greater coordination and cohesiveness (Lucon‐Xiccato et al., 2022) and to escape from predator threats faster (Griffiths et al., 2004; Nadler et al., 2021). Foraging success is higher in familiar than unfamiliar shoals (Ward & Hart, 2005), while familiar individuals are less likely to steal food from each other (Webster & Hart, 2007), discover novel food patches more quickly (Ward & Hart, 2005), and are faster to learn foraging routes from each other (Swaney et al., 2001). Fish associating with familiar individuals have also been shown to experience lower aggression (Utne‐Palm & Hart, 2000), which has been proposed as the driver of the improved growth and condition seen in familiar shoals (Seppä et al., 2001).

Despite the extensive literature on familiarity preferences in multiple fish species, mixed results have been reported regarding the existence and strength of familiarity preferences in zebrafish (*Danio rerio*), the most widely studied fish species. Although initially a model organism for developmental biology and genetics (Choi et al., 2021; Norton & Bally‐Cuif, 2010), the behavior and ecology of *D. rerio* has received increased attention in recent years (Spence et al., 2008; Kalueff et al., 2013; Parichy, 2015). *Danio rerio* have been shown to exhibit shoaling preferences on the basis of different shoal properties (Krause et al., 1999; Pritchard et al., 2001; Velkey et al., 2022), and as a social fish that forms stable shoals in the wild (Shelton et al., 2020) they might be expected to also choose shoals based on familiarity, as documented in other species. While there is some evidence for familiarity preferences in *D. rerio* (Gerlach & Lysiak, 2006; Mukherjee & Bhat, 2023), other studies do not support the existence of familiarity preferences in this species (Blonder & Tarvin, 2022; Santacà et al., 2021) and the reasons for this variation are not clear.

We were interested in evaluating how test design might affect familiarity preferences in *D. rerio*, given these varying reports in the literature. While social preferences vary between species (Santacà et al., 2021; Ward et al., 2009) and familiarity preferences can be affected by both internal and external factors (Benhaïm et al., 2016; Frommen et al., 2007; Griffiths & Magurran, 1997b), variation in test methodology can also be highly influential, and it is essential to optimize methodology and apparatus for robust and reliable studies of behavior (Jones et al., 2023).

A common laboratory method for evaluating social preferences in fish is the three‐chamber, two‐choice shoaling test. A central, focal individual can see and visit two spatially separated social stimuli (e.g., shoals of conspecifics) that differ in one dimension, and the time spent with each stimulus can then be measured and compared. Individuals of social species, including *D. rerio*, will typically avoid being isolated in the center of such a test apparatus, and so the intervening distance between the social stimuli is an important test property: too close together and the focal individual may perceive the two stimuli as essentially being one continuous shoal and so not discriminate between them, too far apart and they may not be able to recognize distinguishing cues from distance. Either of these scenarios would reduce the likelihood of detecting a social preference based on association time.

We therefore ran a series of two‐choice shoaling tests to determine how the intervening distance between a familiar and an unfamiliar shoal affected familiarity preferences in *D. rerio*. Focal subjects experienced three different tests, with stimulus shoals either 30, 45 or 60 cm apart, and were tested on their preference for the two shoals after an initial habituation period. If subjects were better able to recognize and discriminate fish in the stimulus shoals when they were closely spaced, familiarity preferences would be strongest at the shortest distance. If, however, subjects could distinguish individuals at all distances, familiarity preferences would be similar at all distances. They could even be stronger at the longest distance if large open spaces are aversive, as subjects might initially chose to associate with the familiar shoal, and then be more reluctant to cross the open space to visit the other shoal.

## 2 MATERIALS AND METHODS

### 2.1 Animals

All fish in the experiment were mixed‐sex, 12‐month‐old adults from our breeding population of *D. rerio*, originally maintained in groups of eight to 12 individuals from multiple unrelated matings. These groups were housed in 5‐L polycarbonate tanks, each containing a plastic plant, with a constant recirculating supply of water draining to a sump for biological and particulate filtration. At the start of the experiment, 63 individuals were randomly selected from stock groups and moved into one of five 45 × 30 × 30 cm (length × width × height) glass tanks to make new social groups of 11–14 mixed‐sex subjects. A further 20 mixed‐sex individuals, drawn from different groups to the subjects, were moved into a 60 × 30 × 30 cm glass tank to be used as unfamiliar stimulus fish for the experiment. After 6 weeks to allow groups to stabilize and ensure that social familiarity was well established (Pavlidis et al., 2011; Velkey et al., 2022), we started running shoaling tests. All glass tanks contained gravel substrate, three plastic plants, a foam bubble filter, an aquarium heater, and a floating thermometer. Tanks were blacked out on three sides and positioned so that fish could not see into other tanks. Fish were fed daily with Tetramin Flakes fish food, water in all tanks was maintained at 27 ± 1°C, and lights were on a 12 h:12 h day:night cycle. Fish were checked daily for signs of ill‐health such as lethargy, abnormal swimming, weight loss etc. (Reed & Jennings, 2011), and if necessary were euthanized following UK Home Office Schedule 1 methods.

### 2.2 Experimental overview

Subjects were tested on their shoaling preferences in choice tests with a shoal of four familiar individuals from their own tank and a shoal of four unfamiliar individuals from the separate tank of unfamiliar stimulus fish. Tests were carried out in a dedicated testing tank and each subject was tested three times, with the stimulus shoals presented behind transparent partitions with interval distances of 30, 45 or 60 cm between the partitions. The individual identity of each subject was not tracked across the three tests, and so all individuals from a subject tank were tested sequentially at one distance in a single testing session. The order of testing at each distance was randomized for each tank of subjects, and the side on which the familiar shoal was placed was randomized across tests. At least 7 days elapsed between tests for each subject. A small number of subject fish were not tested at each distance due to signs of ill‐health, giving final sample sizes of *n* = 40 for the 30 cm distance, *n* = 43 for 45 cm, and *n* = 42 for 60 cm.

### 2.3 Testing procedure

**FIGURE 1:**
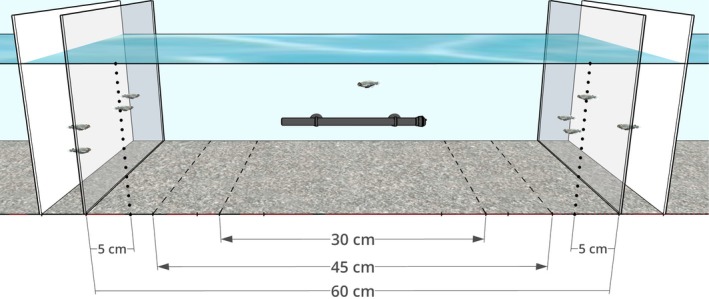
A schematic of the test tank, showing two stimulus shoals of four *D. rerio* behind transparent partitions at each end, and a single focal subject in the central area, with a heater positioned on the rear of the tank. The transparent partitions were either 30, 45 or 60 cm apart, and the time each subject spent within 5 cm of the familiar and unfamiliar shoals was measured, as well as the number of such visits. Dashed lines on the substrate indicate the three possible positions of partitions, while the dotted lines on the front indicate the 5 cm shoaling zones used at the illustrated 60 cm partition spacing.

Shoaling choice tests were conducted in a 120 × 30 × 38 cm glass tank containing 17 cm depth of water at 27°C, an aquarium heater, and gravel substrate. The tank was separated into a central zone for the focal subject, with stimulus shoals presented on the left and right behind clear plastic partitions spaced either 30, 45 or 60 cm apart, with opaque white plastic partitions a further 5 cm behind these to contain the stimulus shoals (Figure 1). At the start of each test, a rectangular 15 × 12 × 25 cm clear plastic tube was placed in the middle of the central zone, and a focal subject was selected at random from their home tank and transferred with a net into the tube. The subject was allowed to habituate for 2 min, then the tube was lifted out to release the focal subject, and their behavior was recorded live for 10 min by an observer (who was not blind to the familiarity status of the shoals) seated 1.5 m in front of the tank who used JWatcher (https://www.jwatcher.ucla.edu) to code subject behavior. Shoaling zones equivalent to approximately two body lengths (Pitcher, 1986) were marked on the front of the tank, 5 cm from the clear partitions. Every entry and exit by a focal subject into either shoaling zone was recorded to calculate the duration of each shoaling visit. Fish were considered to have entered/exited the shoaling zone when at least the front half of the body had crossed into or out of the shoaling zone. Stimulus shoals were formed prior to testing by selecting two males and two females haphazardly from the subjects' tank and transferring them with a net to their designated end zone, after which two males and two females were transferred from the unfamiliar stimulus fish tank and placed in the opposite end zone, and both shoals were given 10 min to habituate to the test tank. The unfamiliar stimulus fish were deliberately chosen to be of similar sizes to the familiar stimulus fish. Both shoals of stimulus fish were changed for each tank of subjects, at each test distance. At the end of each test, the focal subject was moved with a net to a holding tank until all fish in its tank had been tested, at which point all subjects, the familiar shoal fish, and the unfamiliar stimulus fish were returned to their respective home tanks.

### 2.4 Statistical analysis

Data were extracted from JWatcher and analyzed using RStudio v.2023.12.1 (Posit Team, 2024) and R v.4.3.2 (R Core Team, 2023). The total duration and total number of visits to each shoal was calculated for each individual at each test distance. The difference in total time spent with the familiar and unfamiliar shoals was then calculated for each individual at each distance, as was the difference in the number of visits to the familiar and unfamiliar shoals. For all such difference scores, a value of zero indicated equal duration/number of visits to the familiar and unfamiliar shoals, positive values indicated more time/visits with the familiar shoal, and negative values indicated more time/visits with the unfamiliar shoal. The difference scores for duration and the difference scores for number of shoal visits at the 30, 45, and 60 cm intershoal distances were each checked for normality of data and absence of outliers by visual inspection of QQ plots and boxplots. One‐sample *t*‐tests were used (as parametric test assumptions were met) to analyze shoaling duration scores at 45 cm, and shoal visit number scores at 30, 45, and 60 cm. One‐sample Wilcoxon signed rank tests were used (due to the presence of outliers and/or non‐normal data) to analyze shoaling duration difference scores at 30 and 60 cm. For each test, difference data were compared against a hypothetical mean difference of 0. To account for the multiple testing of subject fish, we applied a Holm–Bonferroni correction to the *p* values of the six separate tests we ran. Data and code for all analyses will be available on publication at Zenodo (https://doi.org/10.5281/zenodo.11279218).

## 3 RESULTS

**FIGURE 2:**
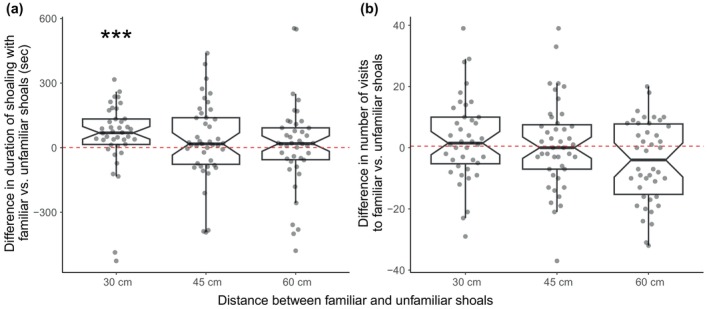
Notched box and whisker plots showing the differences in (a) the duration and (b) the number of shoaling visits by *D. rerio* focal individuals to familiar and unfamiliar conspecific stimulus shoals in choice tests in which the distance between shoals varied. Focal subjects were considered to be shoaling when they entered demarcated zones 5 cm from the clear partition separating each shoal from the central area containing the focal subjects. Central lines indicate medians, boxes extend from the 1st to the 3rd quartile, whiskers show the range up to 1.5× beyond the boxes, and the notches indicate approximate 95% confidence intervals for the medians. Positive values indicate more time/visits with the familiar shoal, negative values indicate more/visits with the unfamiliar shoal, and the dashed red line at each origin represents no difference in behavior towards the two shoals.

At the 30 cm intershoal distance, subjects spent significantly longer (*V* = 658, *p* = 0.003) in proximity to the familiar shoal than the unfamiliar shoal (Figure 2a), spending on average 1.4× longer with the familiar than the unfamiliar shoal (mean ± S.E.M. time with familiar shoal 179.4 ± 14.4 s; time with unfamiliar shoal 125.3 ± 16.3 s). However, subjects did not spend significantly longer with either familiar or unfamiliar shoals at the 45 cm (*t*_42_ = 1.083, *p* = 0.940) or 60 cm distances (*V* = 510, *p* = 0.945).

There was not a significant difference in the number of visits made by subjects to the familiar or unfamiliar shoals at the 30 cm distance (*t*_39_ = 1.206, *p* = 0.940), the 45 cm distance (*t*_42_ = 0.455, *p* = 0.945), or the 60 cm distance (*t*_41_ = −2.180, *p* = 0.175; Figure 2b).

The total time each subject spent in proximity to both shoals was similar at the 30 cm (304.7 ± 16.6 s), 45 cm (283.8 ± 21.8 s), and 60 cm distances (280.9 ± 16.8 s). The total number of visits each subject made to both shoals was highest at the 30 cm distance (61.0 ± 3.9), intermediate at the 45 cm distance (46.4 ± 3.4), and lowest at the 60 cm (35.8 ± 2.7) distance (Figure S1).

## 4 DISCUSSION

In two‐choice shoaling tests with familiar and unfamiliar conspecifics, *D. rerio* subjects showed clear preferences to associate with shoals of familiar individuals that they had shared a tank with for 6 weeks, but this preference was only shown at the shortest intershoal distance of 30 cm. When the familiar and unfamiliar shoals were separated by 45 or 60 cm, this preference was not seen and there was no difference in how much time subjects spent with each other. The number of visits that subjects made to each shoal was not significantly biased to either shoal at any of the distances, indicating that at 30 cm it was the duration of shoaling with familiar fish that was longer, and not that subjects made more visits to the familiar shoal. These results suggest that at the shortest 30 cm distance subject fish were able to evaluate and compare the two shoals, but were potentially unable to compare and distinguish the two shoals at the longer distances. The 45 and 60 cm distances may have been too great for subjects to be able to simultaneously recognize and evaluate the cues of familiarity that they could detect when the shoals were separated by 30 cm, hindering their ability to compare the two shoals. The wider empty space between the shoals at the longer distances may also have inhibited subjects' behavior so that they did not reliably express a preference for familiarity. The increased risk associated with crossing a larger empty space may have deterred subjects from switching to the other shoal, such that the time spent with each shoal was comparable at the 45 and 60 cm distances.

Shoaling preferences in fish are most commonly measured on the basis of time spent with stimulus shoals (Engeszer et al., 2004; Krause et al., 1999; Pritchard et al., 2001), but counts of shoal visits have also been used to assay shoaling behavior (Blonder & Tarvin, 2022; Ghoshal & Bhat, 2021). The number of shoal visits has sometimes been interpreted as a measure of activity (Durrer et al., 2020; Gómez‐Laplaza & Fuente, 2007) or social sampling (Dimitriadou et al., 2019) rather than social preference, and as such it is not necessarily surprising for the two measures to differ in our tests. Other researchers investigating shoaling preferences have reported robust effects when measuring shoaling duration despite finding no difference in the number of visits to different shoals (Cattelan & Griggio, 2018). Indeed, zebrafish have been shown to prefer large shoals over small shoals of conspecifics when shoaling duration is assayed, but not when the number of shoaling visits is compared (Seguin & Gerlai, 2017). When total visits to both familiar and unfamiliar shoals were calculated, we found that subjects visited the stimulus shoals most when the shoals were 30 cm apart, and least when they were 60 cm apart. As the total time with both shoals was similar at the three distances, this suggests that rather than inhibiting shoaling behavior, the larger intershoal distances inhibited switching between shoals, possibly due to the longer empty space being perceived as riskier to cross.

Other researchers working with *D. rerio* have reported mixed results on familiarity preferences, and the variation in cue type used, test area design, and distance between shoals appears to be significant. Wild‐caught zebrafish have been shown to prefer familiar conspecifics in similar choice tests to our own, where shoals were 30 cm apart but presented behind perforated partitions that provided access to visual and olfactory cues (Mukherjee & Bhat, 2023). Familiarity preferences have also been reported from olfactory experiments using a two‐choice odor preference assay in which laboratory‐reared wild‐type zebrafish could swim between adjacent flumes containing familiar and unfamiliar conspecific odor cues (Gerlach & Lysiak, 2006). We deliberately chose a simple design for our shoal choice tests, mimicking a typical design used with many other species in many social preference contexts, in the hope that this would facilitate the detection of a preference for familiarity. Previous research which failed to find familiarity preferences in *D. rerio* employed more complex methodologies and test apparatus, and it may be this that contributed to the reported absence of familiarity preferences. Blonder and Tarvin (2022) tested wild‐type domesticated zebrafish using a 26 cm arena containing maze‐like barriers that subjects had to navigate to reach either stimulus shoal and that only permitted visual access to a single shoal at a time. Santacà et al. (2021) mixed visual and olfactory cues of familiarity in experiments with wild‐type domesticated zebrafish using a test tank in which stimulus shoals were 60 cm apart and found no evidence of familiarity preferences based on odor cues using this protocol. Such test designs would limit the ability of focal subjects to reliably evaluate stimulus shoals simultaneously and given our finding of a clear familiarity preference only at the shortest intershoal distance, *D. rerio* individuals may need close visual or olfactory comparison to be able to evaluate and choose between familiar and unfamiliar shoals.

There is a diversity of social structures in *D. rerio*, with fish living in both large and small shoals in the wild (Shelton et al., 2020; Suriyampola et al., 2015), but familiarity effects are likely to only occur in smaller groups due to limits on the ability of individuals to recognize increasing numbers of conspecifics (Griffiths & Magurran, 1997b; Ward & Hart, 2003). Both males and females form dominance hierarchies (Paull et al., 2010), and there is evidence from dyadic tests that stable social relationships can be established within 5 days in *D. rerio* (Pavlidis et al., 2011). The time it would have taken for familiarity to be established in our housing conditions is likely to have been somewhat longer given the number of individuals in our groups (11–14 individuals per group), and thus we allowed a longer initial period of 6 weeks to give full opportunities for familiarity to form. Whether considering social recognition or familiarity, it is clearly important to consider prior social background and housing experience (Webster & Rutz, 2020), as well as test design.

Both visual and olfactory cues influence social and other behaviors in *D. rerio* (Abreu et al., 2016; Spence et al., 2008), but we focused explicitly on visual cues of familiarity, and the partitions used to separate stimulus shoals from subject fish were not perforated. This limited the passage of olfactory cues from the stimulus shoals to subjects, although partitions were not sealed around the edges and so it may have been possible for chemical cues from the stimulus shoals to reach the central chamber. However, subjects spent little time around the edges of the partitions and spent most of their time at the partitions close to stimulus fish, suggesting that they were not seeking out available conspecific chemical cues. In foraging contexts, *D. rerio* have been shown to rely primarily on visual cues (Howe et al., 2018), and while they have been shown to exhibit small preferences for conspecific odor cues over conspecific visual cues, they do not appear to prioritize olfactory cues over visual cues in a familiarity context (Santacà et al., 2021). Indeed, zebrafish studies in which olfactory cues have been presented at different distances have reported differing results, with familiarity preferences only seen when the familiar and unfamiliar cues are in close proximity (Gerlach & Lysiak, 2006; Santacà et al., 2021), results that mirror our own, in which only visual cues were available. *D. rerio* are known to integrate chemical and visual information (Li, 2019) and it would perhaps be interesting to see if more explicit access to chemical as well as visual cues from conspecific stimulus shoals would enhance familiarity preferences in *D. rerio*.

Our results reinforce the importance of test design in measuring behavior and show that even well‐studied behaviors in a common study species are sensitive to relatively small changes in apparatus or methodology. Two‐choice tests are a robust and commonly used method for testing shoaling in fish, but are also used more widely to measure different behaviors in a range of animal species. Optimizing test design, including the distance at which cues are presented, is essential to ensure robust measurement of preferences in two‐choice tests, and researchers should carefully consider the natural behavior of their study species, the sensory domains they rely on, the choice they are being asked to make, and how best to ensure tests are not unduly aversive. Careful test design, including the use of pilot trials of different configurations, will maximize the reliability and repeatability of results to help improve our understanding of animal decision‐making.

## AUTHOR CONTRIBUTIONS

C.E. and J.P.D. carried out experimental work and data collection. W.T.S and A.R.R. planned the study and wrote the manuscript. W.T.S analyzed the data.

## FUNDING INFORMATION

No specific funding was required for this research.

## Supporting information


Data S1.
