# Supplementary material for: Familiarity preferences in zebrafish (Danio rerio) depend on shoal proximity
Source: J Fish Biol. 2024 Oct 14;107(4):1122–8. doi: 10.1111/jfb.15963 (PMC12536045; doi:10.1111/jfb.15963)
Supplement: Supplementary file 1 — Data S1. [file JFB-107-1122-s001.pdf]

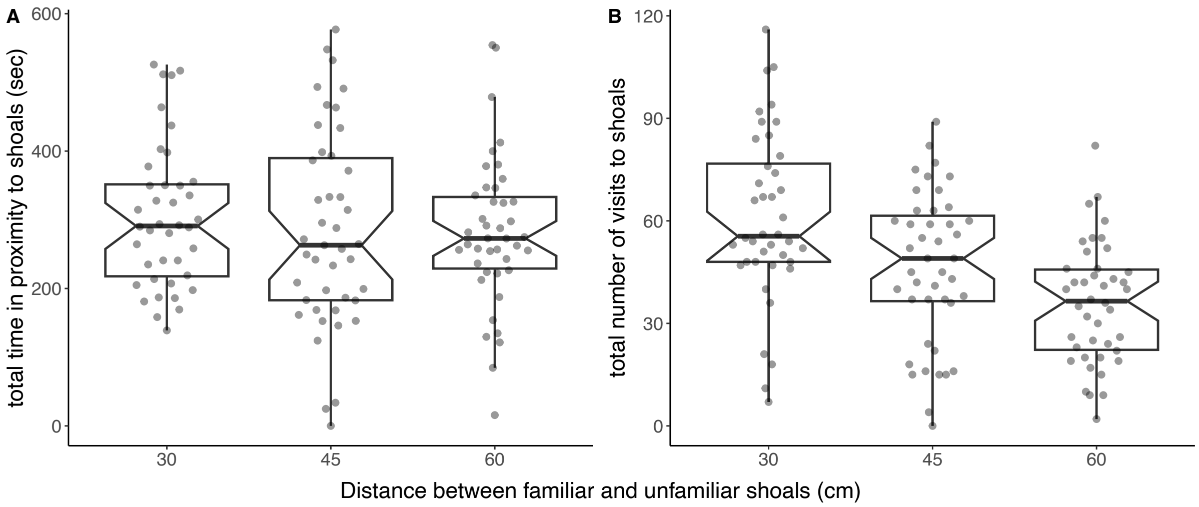

Supporting figure 1. Notched box and whisker plots showing (a) the total duration and (b) the total number of shoaling visits by *D. rerio* focal individuals to both familiar and unfamiliar conspecific stimulus shoals in choice tests in which the distance between shoals varied. Focal subjects were considered to be shoaling when they entered demarcated zones 5cm from the clear partition separating each shoal from the central area containing the focal subjects. Central lines indicate medians, boxes extend from the 1st to the 3rd quartile, whiskers show the range up to 1.5x beyond the boxes, and the notches indicate approximate 95% confidence intervals for the medians.
